# A Paradigm for Matching Waking Events Into Dream Reports

**DOI:** 10.3389/fpsyg.2020.01430

**Published:** 2020-07-03

**Authors:** JiaXi Wang, JingYu He, Ting Bin, HuiYing Ma, Jing Wan, XinQuan Li, XiaoLing Feng, HeYong Shen

**Affiliations:** ^1^School of Psychology, South China Normal University, Guangzhou, China; ^2^School of Psychology, Jiangxi Normal University, Nanchang, China

**Keywords:** actions, attribution, behavior, dreaming, emotions, incorporation, metaphor

## Abstract

In this study, participants recorded their waking events (Personal significant events, PSEs/Major concerns, MCs) and dream reports for 7 days. These events and dreams were paired by the same day (216 PSEs-dreams pairs and 215 MCs-dreams pairs). Then participants were instructed to both find similar features (characters, objects, locations, actions, emotions, and themes) of their events-dreams pairs and give a match score of their events-dreams pairs. Besides, we proposed a method for independent judges to match waking events into dreams (the external ratings). The rating standard of the external-ratings was to look for similar behaviors between events and dreams. Based on this rating standard, three independent judges were instructed to rate participants’ events- dreams pairs. Firstly, we compared the two kinds of methods of self-ratings. Spearman correlations showed that the two methods were significantly correlated with each other. These results suggested that the sum of different kinds of similar features could be used to represent self-ratings reported of the degree of the correlation between a waking event and a dream. Regression correlations showed that for PSEs-dreams pairs, actions, emotions, and themes were similar features that affected the degree of the correlation between an event and a dream of the same day, and for MCs-dreams pairs, emotions, and themes were similar features that affected the degree of the correlation between an event and a dream of the same day. These results suggested that different kinds of similar features had different influence on the self-ratings’ evaluation for the degree of matching between waking event and dream. Secondly, we compared the rating results of the self-ratings and the rating results of the external-ratings. Spearman correlations showed that the results of the self-ratings were significantly correlated with the results of the external-ratings. So this study’s method for the external ratings may be suitable for future studies. Besides, as the external ratings of this study can rate dream metaphors, we also made a short discussion on dream metaphors. Future studies can use the method to explore dream metaphors.

## Introduction

Dreaming is a universal experience that occurs during sleep. There is evidence for a 7-day U-shaped timescale of incorporation of memories of experiences when awake into dreams, in which events from 1 or 2 days before the dream, and from 5 to 7 days before the dream, are preferentially incorporated into dream content (e.g., Nielsen and Powell, 1992; Nielsen et al., 2004; Blagrove et al., 2011; van Rijn et al., 2015; Eichenlaub et al., 2019). The recent incorporations are termed the day-residue effect, and the delayed incorporations the dream-lag effect.

Most of the recent studies concerning the dream-lag effect used similar methods. Participants recorded their waking events and dreams, and then rated correlations between these waking events and dream reports (the self-report method). In addition to the self-report method, some studies also used the independent-judge method (e.g., Blagrove et al., 2011; van Rijn et al., 2015). In this situation, external judges who do not know participants’ information rated correlations between participants’ waking events and dream reports. Evidence showed that when compared with the independent-judge method, the self-report method has the advantage of studying the dream-lag effect (for a discussion, see the introduction section in Eichenlaub et al., 2019). However, in our opinion, when a study aims to explore whether some personality traits can affect conscious experiences’ incorporation into dreams, the independent-judge method is more suitable than the self-report method. The reason is below: when a person finds correlations between waking events and dream reports, these correlations may reflect a person’s belief in or ability to perceive a correlation, rather than the correlations *per se* (for a short discussion see Malinowski, 2015, 13–14). For example, using the questionnaire method, Aumann et al. (2012) found that personality trait variable “openness to experience” was associated with dream variable called “Incorporation (e.g., I dream of people I met the preceding day).” As trait openness is related to imagination, people with high openness may be more able to perceive the correlation between waking experience and dream content, when compared with people with low openness. By contrast, the independent-judge method asked external judges to match all participants’ conscious experiences to dreams, based on operational definitions that have already been formed, and thus it can reduce errors caused by different trait groups, for all the matching would be done by a similar criterion (a same independent judge). So the independent-judge method may be more suitable for this kind of research.

Previously, we developed two operationalized definitions for independent judges to match conscious experiences and dreams, the descriptive-incorporation and the metaphorical-incorporation (Wang and Shen, 2018). The key to this rating method was to judge whether there was a similar behavioral outcome in both a waking event and a dream. We defined the behavioral outcome as the result of a (significant) situation, usually bringing either an advantage (e.g., to fulfill one’s desire, to solve a problem, etc.) or a disadvantage (e.g., to cause a danger, to let someone down, etc.). Yet this definition for the behavioral outcome is vague and thus may lead to methodological errors. In this study, we aimed to revise such criteria for evaluation.

In our opinion, for the judges to match waking events into dream reports in a reliable way, two requirements are needed. Requirement 1: Independent judges should look for similar elements that frequently appear in both waking events and dream reports. Requirement 2: Independent judges’ subjective judgment should be minimized. According to these two standards, in the following, we would try to determine the rating standard for the independent-judge method. Fosse et al. (2003) explored the incorporation of an individual’s waking events into dream reports. In their study, participants identified any element in the dream – characters, objects, actions, locations, emotions, and themes – that seemed likely to have been caused by specific waking events or thoughts from the preceding 2 weeks. The results showed that the most frequent features were themes (53%), emotions (52%), and characters (50%), followed by actions (41%) and objects (39%), with locations having the lowest subject average (29%). Among these features, the rating criteria for judges’ evaluation may be established. On the one hand, based on the above requirement 1, the locations should be excluded from the rating criteria, due to its low frequency. On the other hand, based on the above requirement 2, the emotions and the themes should be excluded from the rating criteria, because in a narrative report (waking events and dreams), to rate them may require more subjective inference than other features. So characters, objects, and actions may be suitable features for independent judges’ evaluation.

Research into the contents of dreams reveals a person’s dreams to be preoccupied with social reality (e.g., Kahn and Hobson, 2005; McNamara et al., 2005; for a review see Revonsuo et al., 2016). Based on previous evidence, Revonsuo et al. (2016) proposed a social simulation theory of dreaming, which defines dreaming as a world-simulation of social perception, cognition, bonding, and social interaction that carries benefits into waking life. This theory was then partly supported by Tuominen et al. (2019), which showed that social situations were more common in dreams than in corresponding waking life. According to the dream continuity hypothesis, dreams express conscious concerns and emotional preoccupations (e.g., Domhoff, 2003, 2011; for a critical review, see Domhoff, 2017). So social situations in a person’s dreams may be correlated with the person’s concerns and preoccupations with social reality in waking life.

Attribution is the process by which individuals explain the causes of behavior and events in waking life (e.g., Kelley and Michela, 1980). In a social event, there were always three elements of behavior: the behavioral subject (doer), the behavioral action (action), and the behavioral target (doee). The doer causes or instigates action, and the doee is the recipient of the action. For example, behavior: He helps her. The doer is he, and the action is the verb help, and the doee is she. Brown and Fish (1983) studied the causality implicit in English verbs that name interactions, either mental or behavioral, between two persons, verbs such as like, notice (mental), and help, cheat (behavioral) in such a context as Ted-Paul. Their results showed that people think of causality in such verbs as unequally apportioned between interactants. For action verbs, greater causal weight is given to the agent argument of the verb (e.g., Ted in Ted helps Paul) than to the Patient argument (Paul). For mental (or state) verbs greater causal weight is given to the Stimulus argument of the verb (e.g., Paul in Ted likes Paul) than to the Experiencer argument (Ted). These results suggest that in an attribution process, the behavioral action will be focused on, while other attention may be paid on either the doer or the doee.

As stated above, dreams may reflect a person’s concerns and preoccupations with social reality in waking life. So in an attribution process, the behavioral action may be more likely to appear in dreams than the behavioral doer and the behavioral doee. As dreams are composed narratives (e.g., Montangero, 2012), and narratives always contain behavioral actions, the behavioral action may be a useful element for the independent-judges method. This method would be better than the method used in Wang and Shen (2018) because judges are more likely to find similar actions of events and dreams literally rather than similar behavioral outcomes of events and dreams literally. Based on this angle, this study tried to use the independent-judge method to match waking events into dreams (for detail, see “Materials and Methods”). For a comparison purpose, we used the self-report method. So in this study, we used both the self-report method (participants rated their own events-dreams) and the independent-judge method to match waking events into dream reports.

Besides, for the self-report method, many studies adopted a similar method as Fosse et al. (2003). Participants identified similar features of events and dreams. Then these features would be used to represent the degree of correlation between a waking event and a dream (e.g., van Rijn et al., 2015). However, it was still unclear whether this way was reliable, because different features of events and dreams may have different influence on participants’ judgment for a correlation in an events-dreams pair. For example, a dreamer may find similar locations in an events-dreams pair, but this does not mean that the dreamer will think the events and the dreams are related, because different things can happen in the same location. A way to solve this problem may be to give a single score to represent the whole correlation in an events-dreams pair, similar to Blagrove et al. (2011) where a six-point Likert scale was used for participants to match their waking events with dreams. A comparison between these two ways of the self-report method may help to show which features can affect the self-ratings’ evaluation for the degree of matching between waking event and dream. So in this study, participants were instructed to rate both similar features of events-dreams and give single scores to represent the correlation between events-dreams.

## Materials and Methods

### Participants

Sixty participants (12 males, 48 females; mean age = 20.37, SD = 1.86, from 18 to 26), all students at South China Normal University, participated in the study. Participants were not taking recreational drugs and alcohol during the period of the experiment; non-smokers; without sleep disorders or neurological/psychiatric history. All subjects gave written informed consent before the start of the study, and this study was approved by the research ethics committee of South China Normal University.

Of the 60 participants, two participants were removed from the analysis because they did not record their waking events and dreams on time every day. Results are thus from the remaining 58 participants (11 males, 47 females; mean age = 20.38, SD = 1.89, from 18 to 26).

### Daily Log

The daily diary was taken from Fosse et al. (2003). It contained three kinds of events, the major daily activities (MDAs), the personally significant events (PSEs), and the major concerns (MCs). To reduce the potential load for ratings, in this study, only PSEs and MCs were recorded. Each evening participants recorded information in the diary about their experiences during the day for the following two categories.

1. Personally significant events (PSEs): Important daily events that may or may not have taken up much time (e.g., emotional events).
2. Major concerns (MCs): Concerns or thoughts that participants had on their mind during the day that may not have taken up much time, but were still considered important to them (e.g., money problems, exam stress).

Up to three items could be recorded in each category. For each item reported, participants were also instructed to rate the intensity of the emotion on a scale from 1 (low) to 5 (high).

### Dream Collection

The method of recording a dream diary was similar to that recommended by Selterman et al. (2012): Describe everything in your dreams, with as much detail as possible: What happened, in what time frame, with whom, etc. Describe the cognitions, emotions, and behaviors you experienced in your dreams, as well as the cognitions, emotions, and behaviors of all other parties included in your dreams (if evident to you). If it was a lucid dream, state so. Continue on the reverse side of this sheet if needed.

### Dream Decoding

#### The Self-Report Method

Participants were instructed to match each waking event and each dream coming from the same day (waking events of a day and that night’s dream), using the following five-point Likert scale: 1. *Absolute no correlation*. 2. *No correlation*. 3. *Unsure correlation*. 4. *Correlation*. 5. *Absolute correlation*.

In addition, participants were instructed to identify any features in the dream – characters, objects, actions, locations, emotion, and themes – that seemed likely to have been caused by specific waking events from the previous day.

#### The Independent-Judge Method

In this study, we used our previous definitions (Wang and Shen, 2018) for representing correlations between waking events and dreams: descriptive-incorporation and metaphorical-incorporation. The idea of this paradigm was taken from Hall and Nordby (1972) who noted that there were two kinds of dreams, denotative dreams and metaphorical dreams. Denotative dreams directly represent their corresponding conscious experience, while metaphorical dreams represent something less obvious and may express complex, even contradictory ideas. Denotative dreams do not require any kind of “decoding” to understand their conscious life referent, whereas metaphorical dreams do. Descriptive-incorporation is the incorporation of conscious experiences into dreams in a direct way. Metaphorical-incorporation is the incorporation of conscious experiences into dreams in an indirect way, which is filled with symbolic expressions. Their operationalized definitions are in Table 1.

**TABLE 1:** Operational definitions of different kinds of incorporation.

Category	Operational definition
Descriptive-incorporation^a^	There are similar behavioral actions in the waking event and the dream, and these similar behavioral actions are done by the same behavioral subject (doer), and these similar behavioral actions are toward the same behavioral target (doee).
Metaphorical-incorporation^a^	There are similar behavioral actions in the waking event and the dream. But these behavioral actions are not done by the same behavioral subject (doer), or these behavioral actions are not toward the same behavioral target (doee).

*^*a*^In this study, the adjective similar means that two elements can be categorized as of the same taxonomy, in which the taxonomy can be seen from recording literally. This is because the two elements refer to the same thing, for example, for a case of similar action, event: go shopping; dream: go to Wal-Mart. For a case of similar character, event: I was waiting for the result of tutor selection; dream: my favorite tutor told me that I was selected (Note that in this case tutor selection contains my favorite tutor’s selection, so both the event and the dream were viewed as having similar characters).*

### Procedure

Participants were asked to record their dreams and waking events in a spreadsheet at home. As a reward, they could get feedback about their dream reports and monetary compensation. Participants were instructed to record events-dreams for 7 days. Specifically, participants recorded their waking events each evening and recorded their night dreams each morning (if they did not have dreams, they were required to record the words: no dreams), via a Chinese online questionnaire resource Wenjuanxing (similar to the online questionnaire resource Qualtrics). In addition, as with the daily diary, they were also instructed to rate the intensity of the emotion on a scale from 1 (low) to 5 (high). Dream diaries and waking experiences were paired by the same day, regardless of their respective numbers (e.g., if four events were recorded in the conscious time and two dreams were reported in a single night, they were counted as one paired event-dream). Then approximately 1 week later, participants were sent materials (via email) so that they could perform the correspondence identification task. Participants were instructed to compare each of their waking events with each dream of the same pair, to identify similar features between the diary items and dream reports, such as the characters, objects, actions, locations, emotions, or themes. Participants were instructed to give a score that represented the correlation between each waking event and each dream of the same pair (for detail, see section “Dream Decoding”). In addition, three independent judges who were blind to participants’ information rated participants’ recordings. The three judges were all co-authors and were all psychological postgraduates at South China Normal University. They scored each type of incorporation for participants’ events-dreams pairs (PSEs-dreams pairs and MCs-dreams pairs) by operational definitions in Table 1. Note that in this study, an events-dreams pair can be rated as more than one kind of incorporation (e.g., both descriptive-incorporation and metaphorical-incorporation), as 1 day may have more than one event. Finally, judges counted the number of events (PSEs/MCs), and the length of dreams in each events-dreams pair.

### Data Analysis

Before the ratings, dream diaries and waking experiences were paired by the same day (for detail, see section “Dream Collection”). Three dreams (from two participants) were not longer than the minimum of 20 words. So data from these three events-dreams pairs were not included in the analysis. Finally, we got 216 paired PSEs-dreams and 215 paired MCs-dreams (one participant only reported PSEs and dreams in 1 day). On average, each participant reported 3.72 events-dreams pairs, with a range of 3–7.

For the self-report method, matching scores for each of the diary event (both PSEs and MCs) to dreams were used. As commonly there would be more than one score in an events-dreams pair, in this study, the highest score of an events-dreams pair (PSEs-dreams pair/MCs-dreams pair) was used. We termed the score as Matching-Score. We summed the total number of similar features between the event (the highest score one) and the dream. We termed the number as Similar-Feature. If there was more than one event having the highest score in an events-dreams pair, we used the average number of similar features between the events and the dreams. In addition, we termed the emotionality of the event (the highest score one) as Event-Emotionality. Similarly, if there was more than one event having the highest score in an events-dreams pair, we used the average number of emotionality of the events. The reason why we used the highest score of each events-dreams pair was to avoid a potential floor effect for further comparisons. Besides, for the Matching-score, the Similar-Feature, and the Event-Emotionality separately, we summed each participant’s rating scores of one’s own events-dreams pairs and divided them by each participant’s reported of the number of events-dreams pairs to get the average number of each kind of the rating score. Then for each participant’s events-dreams pairs, the averaged Matching-score, the averaged Similar-Feature, and the averaged Event-Emotionality were used for further correlation analysis.

For the independent-judge method, three independent judges scored each type of incorporation for the total 216 paired PSEs-dreams and the 215 paired MCs-dreams. If an events-dreams pair was scored as neither descriptive-incorporation nor metaphorical-incorporation, the pair would be scored as non-incorporation, meaning that there was no correlation of an events-dreams pair. The Cronbach’s consistency coefficient was from 0.68 (metaphorical-incorporation) to 0.82 (non-incorporation). All inconsistent ratings were discussed later carefully until reaching an agreement. For these three kinds of incorporations, a score of 0 for no presence or 1 for presence was used. Similar to the self-report method, for each participant’s events-dreams pairs, we calculated out the averaged number of each kind of incorporation. Next, the averaged number of non-incorporation, the averaged number of descriptive-incorporation, and the averaged number of metaphorical-incorporation were used for further analysis. We used Spearman correlations to make comparisons between the self-ratings and the external-ratings.

All statistical analysis was performed in IBM SPSS 18.0 software.

## Results

The analyzed data set consisted of a total of 216 PSEs-dreams pairs and 215 MCs-dreams pairs (from 58 participants). For the 216 PSEs-dreams pairs and 215 MCs-dreams pairs, the average length of dreams (words) was 143.06, SD = 132.99, from 27 to 736. The average number of PSEs for all PSEs-dreams pairs was 2.82, SD = 0.49. The average number of MCs for all participants’ MCs-dreams pairs was 2.67, SD = 0.63. The average Event-Emotionality for all participants’ PSEs was 3.52, SD = 1.11. The average Event-Emotionality for all participants’ MCs was 3.21, SD = 1.12.

In the following, all averaged scores of events-dreams pairs referred to means across participants (rather than individual events-dreams pairs).

### Self-Report Method

For the self-ratings, the percentage of dream report to diary record matchings that were scored as three (unsure correlation) were PSEs, 24.5%; MCs, 20%. The percentage of dream report to diary record matchings that were scored as at least four (correlation) were PSEs, 41.2%; MCs, 25.5%. For similar features of PSEs and dreams, the most frequent features were emotions (43.5%), actions (38.9%), followed by characters (34.7%), themes (31.9%), and locations (30.6%), with objects having the lowest subject average (20.4%). For similar features of MCs and dreams, the most frequent features were emotions (29.3%), followed by actions (21.4%), themes (17.1%), characters (14%), locations (12.1%), with objects having the lowest subject average (5.6%).

Because the data for events-dreams pairs were non-normal, we used the Spearman correlation to compare the correlation between averaged Matching-score and averaged Similar-Feature. For each participant’s PSEs-dreams pairs, a Spearman correlation showed that the averaged Matching-score was significantly correlated with the averaged Similar-Feature (*r* = 0.730, *p* < 0.001). Similarly, for each participant’s MCs-dreams pairs, a Spearman correlation showed that the averaged Matching-score was significantly correlated with the averaged Similar-Feature (*r* = 0.787, *p* < 0.001).

Further, we performed the regressions analysis to examine the prediction of the averaged Matching-score by the predictor variables different kinds of averaged similar features of events-dreams pairs (characters, objects, locations, actions, emotions, themes), and the averaged Event-Emotionality (for detail see Table 2). For PSEs-dreams pairs, the predictor variables explained 55.6% of the variance (*R*^2^ = 0.556; *F*[7,50] = 11.18, *p* < 0.001). For MCs-dreams pairs, the predictor variables explained 66% of the variance (*R*^2^ = 0.66; *F*[7,50] = 16.8, *p* < 0.001).

**TABLE 2:** Regression analysis for the averaged Matching-score as predicted by the different kinds of averaged similar features of events-dreams pairs (characters, objects, locations, actions, emotions, themes), and the averaged Event-Emotionality.

	Averaged matching-score			
	PSEs-dreams pairs^a^		MCs-dreams pairs^a^	
	β	*p*	β	*p*
Characters	0.422	0.132	0.926	0.086
Objects	0.218	0.565	–0.443	0.493
Locations	0.673	0.057	0.906	0.124
Actions	0.798	0.014	0.233	0.623
Emotions	1.03	0.001	1.198	<0.001
Themes	0.875	0.020	1.680	<0.001
Averaged Event-Emotionality	–0.055	0.632	0.25	0.021

*(a) across participants (rather than across each events-dreams pair).*

### Independent-Judge Method

For the external-ratings, the percentage of different kinds of incorporation were: the non-incorporation, for PSEs 70.8%, for MCs 86.5%; the descriptive-incorporation, for PSEs 5.1%, for MCs 6.5%; the metaphorical-incorporation, for PSEs 24.1%, for MCs 7%.

For the non-incorporation, there were significant negative correlations between the self-ratings and the external-ratings (*r* from −0.414 to −0.542, all *p* < 0.001). For the descriptive-incorporation, there were significant positive correlations between the self-ratings and the external-ratings (*r* from 0.275 to 0.465, all *p* < 0.05). For the metaphorical-incorporation, there were significant positive correlations between the self-ratings and the external-ratings (*r* from 0.269 to 0.365, all *p* < 0.05). The detail is shown in Table 3.

**TABLE 3:** The correlation between the self-ratings and the external-ratings^a^.

	PSEs-dreams pair				MCs-dreams pair			
	Averaged matching-score		Averaged similar-feature		Averaged matching-score		Averaged similar-feature	
Averaged non-incorporation	*r*	*p*	*r*	*p*	*r*	*p*	*r*	*p*
	−0.414	0.001	−0.542	<0.001	−0.517	<0.001	−0.511	<0.001
Averaged descriptive incorporation	*r*	*p*	*r*	*p*	*r*	*p*	*r*	*p*
	0.295	0.024	0.275	0.037	0.465	<0.001	0.408	0.001
Averaged metaphorical incorporation	*r*	*p*	*r*	*p*	*r*	*p*	*r*	*p*
	0.269	0.041	0.399	0.002	0.282	0.032	0.361	0.005

*(a) across participants (rather than across each events-dreams pair).*

Tables 4 and 5 show examples of descriptive-incorporation and metaphorical-incorporation.

**TABLE 4:** The example of descriptive-incorporation in an events-dreams pair, for PSEs-dream pair and MCs-dream pair separately.

PSEs		I went to the rooftop to see the sky
Dream		In the evening, I went to the rooftop to see the sky, and.
Analysis	action	go somewhere and see something
	doer	I
	doee	rooftop and sky
MCs		I was waiting for the result of mentor selection of mine
Dream		In my dorm, I received an email from my desired tutor, he said he was satisfied with me, and I felt very happy.
Analysis	action	select student
	doer	my desired tutor
	doee	I

**TABLE 5:** The example of metaphorical-incorporation in an events-dreams pair, for PSEs-dream pair and MCs-dream pair separately.

PSEs		I felt sad for a woman being molested when I saw a documentary.
Dream		I walked on a road, and suddenly I was molested by a stranger.
Analysis	action	being molested
	doer	Event: woman; Dream: I
	doee	
MCs		I am concerned about the final exam for mathematics tomorrow
Dream		After an exam, I went back to my dormitory and found that I failed my C-language exam and my English exam. This made me very sad.
Analysis	action	test for final exams
	doer	
	doee	Event: mathematics; Dream: C-language, English

## Discussion

For the self-report method, for both PSEs-dreams pairs and MCs-dreams pairs, the self-ratings’ averaged Matching-score was significantly correlated with the averaged Similar-Feature. Further regression analysis showed that for PSEs-dreams pairs, actions, emotions, and themes were similar features that affected the averaged Matching-score, and for MCs-dreams pairs, emotions and themes were similar features that affected the averaged Matching-score. These results cannot be explained by the different frequencies of these features. For example, in PSEs-dreams pairs, though themes (31.9%) and locations (30.6%) had similar frequencies, themes were similar features that affected the Matching-score, but not for locations. Similarly, in MCs-dreams pairs, though themes (17.1%) and actions (21.4%) had similar frequencies, themes were similar features that affected the Matching-score, but not for actions. These results may suggest that different kinds of similar features had different influence on the self-ratings’ evaluation for the degree of matching between waking event and dream. Nevertheless, as results showed that the two methods of the self-ratings related to each other, the sum of different kinds of similar features could be used to represent self-ratings’ report of the degree of the correlation between waking events and dreams.

For the independent-judge method, nearly 29.2% of dream reports were found to relate to PSEs, and nearly 13.5% of dream reports were found to relate to MCs. These numbers were lower than Wang and Shen (2018), where nearly 54.7% of dream reports were found to relate to waking events. In the present study, the method of the external ratings was to look for similar behaviors between waking events and dreams. By contrast, in that study (Wang and Shen, 2018), except for similar behaviors between waking events and dreams, the method of the external ratings was also to look for similar behavioral outcomes (such as emotions and cognitive benefits) between waking events and dreams. So in that study, the external ratings found more incorporations of waking events into dreams. The advantage of this study’s external ratings was that it had a higher inter-raters’ consistency (from 0.68 to 0.82, for each incorporation separately), by contrast in Wang and Shen (2018) the inter-raters consistency was 0.74 for all incorporations together. These results may suggest the method of external ratings reported here was better than the rating method of that study. Nevertheless, as in the data analysis process, all inconsistent results of different external raters would be discussed until reaching an agreement, and both the two rating standards (similar behaviors/similar behavioral outcomes) could be used for future studies.

Besides, there were significant correlations between the self-ratings and the external-ratings. These results, to some extent, supported the reliability of the rating standard for the independent-judge method reported here. Many researchers subscribe to the notion that dreams can be metaphors for waking life, picturing waking-life experiences and emotions in non-literal, figurative ways (e.g., Jung, 1948a,b; Lakoff, 1993; for a short review, see Malinowski and Horton, 2015, p10). In this study, the metaphorical incorporation describes the situation that dreams reflect waking events indirectly. In other words, the metaphorical incorporation relates to dream metaphors. Bowdle and Gentner (2005) proposed that metaphors can be seen as a species of analogy. According to the structure-mapping theory (Gentner, 1983), interpreting a metaphor involves two interrelated mechanisms: alignment and projection. The alignment process operates to create a maximal structurally consistent match between two representations that observes one-to-one mapping and parallel connectivity (Falkenhainer et al., 1989). That is, each element of one representation can be located in correspondence with at most one element of the other representation, and arguments of aligned relations and other operators are themselves aligned. Once a structurally consistent match between the target and base representations has been found, further elements from the base that are connected to the common system can be projected to the target as candidate inferences. According to the structure-mapping theory and other analogical accounts, metaphors typically convey that a system of relations holding among the base objects also holds among the target objects, regardless of whether the objects themselves are intrinsically similar (e.g., Gentner and Markman, 1997). In this study, for the external ratings, the rating standard was to look for similar actions between a waking event and a dream report. As has been stated in the introduction section, a behavior contains three elements: the element doer, the element action, and the element doee. In a behavior, the element action plays the role to convey a relationship between the element doer and the element doee. When there are two similar behavioral actions between two events, a metaphor may be established, because the two actions may convey a system of relations holding among one event’s doer and doee also holds among the other one event’s doer and doee. According to Domhoff’s dream continuity hypothesis (e.g., Domhoff, 2017), dreams reflect a person’s waking concerns and preoccupations. So if there are similar behavioral actions between a person’s waking event and the person’s dream, the action in the person’s dream can be seen as reflecting the person’s concern for the similar action in the waking time. As a result, similar actions may convey a system of relations holding among the event’s doer and doee also holds among the dream’s doer and doee. According to the rating standard of the present study, the event and the dream will be rated as either the descriptive incorporation or the metaphorical incorporation. Future studies can use this method of external ratings to judge dream metaphors.

### Limitation

In the introduction section, we proposed that dreams may reflect people’s attribution process, and we viewed that behavioral action can be a key for independent judges to match waking events into dreams. Similar actions of waking events and dreams may be hard for judges to find in some situations: 1. For MCs, people may not record any behavioral action of the event, and rather, they may record a topic and emotions toward this topic (e.g., I am anxious about the final exam). Under this situation, judges may have to imagine an action for these MCs (e.g., I should not fail in the final exam), and then match these MCs into dreams. 2. For PSEs, people may record a behavioral action of the event. But judges may not know this action is related to a topic (e.g., for the event I went to the Wal-Mart, judges may not know the Wal-Mart is a supermarket). Under this situation, though there may be a similar action of the topic in a dream, judges cannot rate out it. Future studies should use both the self-report method and the independent-judge method to do the matching work.

### Directions for Future Research

As we have suggested, similar behaviors can be used to judge correlations between waking events and dreams. Studies on the dream content suggest that there are typical dream themes defined as dreams with similar content reported by a high percentage of dreamers (e.g., Maggiolini et al., 2010; Mathes et al., 2014). To measure typical dreams, Nielsen et al. (2003) developed the Typical Dream Questionnaire (TDQ) with 56 items. Most of these items contained actions. Future studies can try to explore whether there are typical dream actions.

## Conclusion

In this study, we compared two kinds of self-ratings to rate correlations between waking events and dreams. The first one was to sum the number of similar features between a waking event and a dream, and the second one was to give a whole score to represent the correlation between a waking event and a dream. Results showed that these two kinds of methods significantly correlated with each other. So these results suggested that the sum of different kinds of similar features could be used to represent self-ratings reported of the degree of the correlation between a waking event and dream. Besides, we found that for PSEs-dreams pairs, actions, emotions, and themes were similar features that affected the averaged Matching-score, and for MCs-dreams pairs, emotions and themes were similar features that affected the averaged Matching-score. These results may suggest that different kinds of similar features had different influence on the self-ratings’ evaluation for the degree of matching between waking event and dream.

In addition, we proposed a new rating standard for independent judges to do the external-ratings. Results showed that there were significant correlations between the self-ratings and the external-ratings. These results may suggest the reliability of the external-ratings reported here. Future studies could use this study’s methods of external-ratings for more exploration.

## Data Availability Statement

All datasets generated for this study are included in the article/supplementary material.

## Ethics Statement

The studies involving human participants were reviewed and approved by the Ethics Committee in South China Normal University. The patients/participants provided their written informed consent to participate in this study.

## Author Contributions

JXW, HS, and XF: design. JXW, JW, and XL: data collection. JW, JH, HM, and TB: data analysis. JXW: manuscript and revise. All authors contributed to the article and approved the submitted version.

## Conflict of Interest

The authors declare that the research was conducted in the absence of any commercial or financial relationships that could be construed as a potential conflict of interest.
