# Corrigendum: A Paradigm for Matching Waking Events Into Dream Reports

**DOI:** 10.3389/fpsyg.2021.702950

**Published:** 2021-06-04

**Authors:** JiaXi Wang, JingYu He, Ting Bin, HuiYing Ma, Jing Wan, XinQuan Li, XiaoLing Feng, HeYong Shen

**Affiliations:** ^1^School of Psychology, South China Normal University, Guangzhou, China; ^2^School of Psychology, Jiangxi Normal University, Nanchang, China

**Keywords:** actions, attribution, behavior, dreaming, emotions, incorporation, metaphor

In the original article, there was an error in the abstract section, with an inaccurate expression for some results of this research.

A correction has been made to the abstract. The corrected abstract is shown below:

In this study, participants recorded their waking events (Personal significant events, PSEs/Major concerns, MCs) and dream reports for 7 days. These events and dreams were paired by the same day (216 PSEs-dreams pairs and 215 MCs-dreams pairs). Then participants were instructed to both find similar features (characters, objects, locations, actions, emotions, and themes) of their events-dreams pairs and give a match score of their events-dreams pairs. Besides, we proposed a method for independent judges to match waking events into dreams (the external ratings). The rating standard of the external-ratings was to look for similar behaviors between events and dreams. Based on this rating standard, three independent judges were instructed to rate participants' events-dreams pairs. Firstly, we compared the two kinds of methods of self-ratings. Spearman correlations showed that the two methods were significantly correlated with each other. These results suggested that the sum of different kinds of similar features could be used to represent self-ratings reported of the degree of the correlation between a waking event and a dream. Regression correlations showed that for PSEs-dreams pairs, actions, emotions, and themes were similar features that affected the degree of the correlation between an event and a dream of the same day, and for MCs-dreams pairs, emotions, and themes were similar features that affected the degree of the correlation between an event and a dream of the same day. These results suggested that different kinds of similar features had different influence on the self-ratings' evaluation for the degree of matching between waking event and dream. Secondly, we compared the rating results of the self-ratings and the rating results of the external-ratings. Spearman correlations showed that the results of the self-ratings were significantly correlated with the results of the external-ratings. So this study's method for the external ratings may be suitable for future studies. Besides, as the external ratings of this study can rate dream metaphors, we also made a short discussion on dream metaphors. Future studies can use the method to explore dream metaphors.

The authors apologize for this error and state that this does not change the scientific conclusions of the article in any way. The original article has been updated.

